# Effects of Dual‐Task and Single‐Task Interventions on Physical and Cognitive Function in Older Adults: A Scoping Review

**DOI:** 10.1155/jare/9242629

**Published:** 2026-01-07

**Authors:** Deepak Thazhakkattu Vasu, Tammy Pan Jia Yee, Li-Wei Chou, Fong Lai Yen

**Affiliations:** ^1^ Department of Physiotherapy, M. Kandiah Faculty of Medicine and Health Sciences, Universiti Tunku Abdul Rahman, Sungai Long Campus, Kajang, Malaysia, utar.edu.my; ^2^ Department of Physical Therapy and Assistive Technology, National Yang Ming Chiao Tung University, Taipei, 112304, Taiwan (ROC), nctu.edu.tw

**Keywords:** aging, balance, cognitive–motor, dual-task interventions, elderly

## Abstract

**Background:**

Dual‐task and single‐task interventions are strategies to enhance physical and cognitive function in older adults, but their effectiveness in comparison is not well understood. Understanding their effects on cognitive benefits, balance, and overall physical performance is important for developing effective interventions to support aging populations.

**Objective:**

This scoping review aims to identify and synthesize current evidence on dual‐task and single‐task interventions for older adults, categorizing intervention types, study populations, and measured outcomes.

**Methods:**

PubMed, MEDLINE, Scopus, and Web of Science databases were searched. A comprehensive literature search identified studies involving dual‐task and single‐task interventions from 2014 to July 2024 involving adults aged 60 and older. The methodological quality of the included studies was assessed using the Mixed‐Methods Appraisal Tool (MMAT). Studies were categorized based on task type: motor–cognitive dual‐task, motor–motor dual‐task, single‐motor task, or single cognitive task. Extracted data included intervention characteristics, outcome measures, and key findings.

**Results:**

A total of 31 studies met the inclusion criteria with participants being community‐dwelling older adults aged 60 and above. Intervention durations ranged from 6 weeks to 12 months, with most studies implementing sessions 2‐3 times per week. Outcome measures commonly included assessments of balance, cognitive function, and gait performance. Dual‐task interventions demonstrated greater improvements in gait, dynamic balance, and cognitive function compared to single‐task interventions with motor‐cognitive dual‐task training being particularly effective in enhancing balance and mobility in older adults.

**Conclusion:**

This review provides a comprehensive comparison of dual‐task and single‐task interventions, highlighting the superior efficacy of dual‐task training in improving both cognitive and physical outcomes. While single‐task interventions offer benefits, they lack the comprehensive improvements observed in dual‐task training. Future research should explore long‐term outcomes, refine intervention protocols, and assess the applicability of combined approaches to maximize benefits for aging populations. And, studies must prioritize reporting effect sizes and minimum clinically important differences (MCID) to ensure findings are clinically relevant.

## 1. Introduction

The global population is aging rapidly, with projections indicating that by 2030, one in six people worldwide will be aged 60 and above [1]. By 2050, the population of older adults is expected to reach 2.1 billion, highlighting an urgent need to address age‐related health challenges. Aging is associated with declines in both physical and cognitive functions, leading to increased risks of chronic diseases, mobility limitations, falls, and psychological issues such as anxiety, depression, and social isolation [2, 3].

Maintaining physical activity is crucial for older adults to reduce age‐related declines. Regular exercise enhances cardiovascular health, muscle strength, and cognitive function while reducing fall risk [4, 5]. Strength training improves muscle mass and balance, whereas aerobic activities like walking or cycling support endurance and mobility. Despite these benefits, many older adults remain physically inactive due to health constraints or lack of motivation, emphasizing the need for tailored exercise programs that are both engaging and effective [5].

Falls among older adults showed a significant health concern, often leading to injuries, reduced independence, and reduced the quality of life. Balance and mobility impairments, coupled with cognitive decline, contribute to an increased risk of falls [6]. Research has shown that cognitive processes such as executive function and attention play a crucial role in fall prevention. Deficits in these areas can impair an individual’s ability to navigate their environment safely, further increasing fall risk [7].

Dual‐task training (DTT) has emerged as a promising intervention to enhance both cognitive and motor functions simultaneously. Unlike single‐task training, which focuses solely on either cognitive or physical tasks, DTT requires individuals to engage in both, such as walking while solving arithmetic problems [8]. This approach challenges cognitive–motor integration, improving gait stability, balance, and executive function. Various studies have demonstrated that DTT is more effective in reducing fall risks and enhancing overall functional performance compared to single‐task training [9, 10]. Research on DTT remains inconsistent regarding optimal intervention strategies, long‐term effects, and applicability across different populations. Standardization of protocols and outcome measures is essential to maximize its benefits.

This scoping review aims to explore the current literature on dual‐task and single‐task interventions for managing balance and preventing falls in older adults. While study in this area continues to explore, there remains uncertainty about their comparative effectiveness strategies for implementation. The objective is not to determine the effectiveness but to provide an overview of the current research.

Comparing dual‐task and single‐task training in previous review, this review will give an updated overview of current research. It will identify gaps and suggest future directions for improving balance in older adults. The Mixed‐Methods Appraisal Tool (MMAT) will be used to assess the quality of the studies, ensuring a balanced evaluation of quantitative approaches.

## 2. Methods

### 2.1. Eligibility Criteria

The eligible studies for our study were identified using the population, intervention, comparison, outcome, study (PICOS) design approach.

• Population: community‐dwelling adults aged 60 and above, including those using mobility aids but excluding individuals with significant cognitive impairments, neurological disorders, or comorbidities limiting mobility.
• Intervention: dual‐task interventions (motor–cognitive or motor–motor) and single‐task interventions (motor or cognitive).
• Comparison: all interventions
• Outcomes: reported impacts on balance, cognitive function, and overall performance.
• Study design: RCTs were the primary focus, and quasi‐experimental studies were also included to ensure comprehensive coverage of existing literature, consistent with the nature of a scoping review

This review examines interventions for community‐dwelling, sedentary adults aged 60 and older who maintain independent mobility in daily activities, including those who use assistive devices. The target population consists of typically healthy older adults who exercise less than 150 min weekly. While individuals using mobility aids are included, those with significant cognitive impairment, neurological disorders, or mobility‐limiting comorbidities are excluded from the scope of this review. The analysis encompasses various intervention types, including dual‐task interventions combining cognitive and motor elements, motor–cognitive training programs, motor–motor interventions, and single‐task approaches focusing on either motor or cognitive components. These interventions will be evaluated against each other and control groups (no intervention) to determine their comparative effectiveness in fall prevention, fall risk reduction, and balance improvement among older adults. The review will analyze randomized controlled trials (RCTs) published in English.

### 2.2. Search Strategy

To identify the potentially relevant papers, the databases were searched from 2014 to July 2024, and a literature search was performed using PubMed, MEDLINE, Scopus, and Web of Science. The literature search was performed using the following keywords combined with Boolean operators AND and OR: dual task, cognitive‐motor, balance, postural control, stability elderly, older adults, aging, ageing, intervention, training, motor‐motor training, cognitive‐based, cognitive training, cognitive exercise, motor‐based, motor training, and physical exercise.

### 2.3. Study Selection

Two researchers independently reviewed the abstract of each study for the scoping review (T.P.J.Y. and L‐W.C.). Each study’s eligibility was assessed according the inclusion and exclusion criteria, and the results were compared. After completing the search, applicable studies were reviewed, and a consensus was reached regarding study inclusion. In cases of disagreement, a repeated review was conducted, and a third researcher (D.T.V.) was consulted.

This review was conducted in accordance with the Preferred Reporting Items for Systematic Reviews and Meta‐Analyses extension for Scoping Reviews (PRISMA‐ScR) guidelines.

### 2.4. Data Extraction

Data were extracted using a standardized charting form, including author, year, country, study design, sample size and characteristics, intervention type, frequency/duration, outcome measures, and key findings.

### 2.5. Study Quality Assessment

This scoping review was assessed using the MMAT Version 2018 developed by Hong and colleagues [11]. The MMAT is designed to evaluate the methodological quality of the studies across five categories of study designs. Although the tool is intended for mixed studies reviews that may include qualitative, quantitative, and mixed‐methods research, only the criteria relevant to RCTs (Category B) and nonrandomized studies (Category C) were applied in this review, as no qualitative, descriptive, or mixed‐methods studies were included.

The MMAT does not generate a single overall score; instead, it provides criterion‐specific ratings that help inform the methodological quality and interpretation of the included studies. For the purpose of providing a quick visual representation of quality in Table 1, an MMAT^∗^ star rating was assigned to each study based on the number of “Yes” responses across the relevant criteria. This rating system is defined as follows:

 The symbol ^∗^ indicates very low quality
 The symbol ^∗∗^ indicates low quality
 The symbol ^∗∗∗^ indicates moderate quality
 The symbol ^∗∗∗∗^ indicates good quality
 The symbol ^∗∗∗∗∗^ indicates high quality

**Table 1:** Mixed‐methods appraisal tools (MMAT).

No.	First author	Year	Screening questions		Randomized controlled trials					Nonrandomized studies					Quantitative descriptive studies					^∗^MMAT scoring
			S1	S2.	2.1.	2.2.	2.3.	2.4.	2.5	3.1.	3.2.	3.3.	3.4.	3.5.	4.1.	4.2.	4.3.	4.4.	4.5.	
1	Blackwood J	2015	Y	Y	Y	Y	N	U	Y						Y	U	Y	N	Y	^∗∗∗^
2	Smith‐Ray R. L.	2014	Y	Y	Y	Y	N	N	Y						Y	U	Y	N	Y	^∗∗∗^
3	Ten Brinke L. F.	2019	Y	Y	Y	Y	Y	Y	U						Y	Y	Y	U	Y	^∗∗∗∗^
4	Wollesen B.	2015	Y	Y	Y	U	U	N	U						Y	U	Y	U	Y	^∗∗^
5	Wollesen B.	2017	Y	Y	Y	N	U	U	U						Y	U	Y	N	Y	^∗∗^
6	Yuzlu V.	2021	Y	Y	Y	Y	Y	Y	Y						Y	Y	Y	Y	Y	^∗∗∗∗∗^
7	Piotrowska J.	2020	Y	Y	U	U	Y	U	Y						U	Y	Y	Y	U	^∗∗∗^
8	Trombini‐Souza F.	2023	Y	Y	Y	Y	N	Y	U						Y	U	Y	N	Y	^∗∗∗^
9	Nahand M. S.	2020	Y	Y						U	Y	U	N	Y	Y	U	Y	U	Y	^∗∗^
10	Jehu D. A.	2016	Y	Y	U	Y	U	N	Y						Y	U	Y	U	Y	^∗∗∗^
11	Alves de Oliveira V. M.	2020	Y	Y	Y	U	Y	Y	U						Y	U	Y	U	Y	^∗∗∗^
12	Javadpour S.	2021	Y	Y	U	Y	Y	U	Y						Y	U	Y	Y	Y	^∗∗∗^
13	Park J. H.	2022	Y	Y	Y	U	U	Y	U						Y	U	Y	U	Y	^∗∗∗^
14	De Maio Nascimento M.	2023	Y	Y	Y	U	U	Y	Y						Y	U	Y	U	Y	^∗∗∗^
15	Ogawa E. F.	2021	Y	Y	Y	U	Y	U	Y						Y	U	Y	U	Y	^∗∗∗^
16	Jehu D. A.	2016	Y	Y	Y	U	Y	N	Y						Y	U	Y	U	Y	^∗∗∗^
17	Castillo de Lima V.	2023	Y	Y	Y	Y	N	U	Y						Y	U	Y	U	Y	^∗∗∗^
18	Fraser S. A.	2017	Y	Y	Y	U	N	U	N						Y	U	Y	U	Y	^∗∗^
19	Nayak A.	2021	Y	Y	Y	Y	Y	U	Y						Y	U	Y	U	Y	^∗∗∗^
20	Sokołowska B.	2024	U	U	Y	Y	U	U	Y						U	U	Y	U	Y	^∗∗^
21	Eggenberger P.	2015	Y	Y	Y	Y	N	N	Y						Y	U	Y	Y	Y	^∗∗∗^
22	Scarmagnan G. S.	2024	Y	Y	N	N	U	Y	U						Y	U	Y	U	Y	^∗∗^
23	Akin H.	2021	Y	Y	Y	U	U	Y	U						Y	U	Y	U	Y	^∗∗∗^
24	Takeuchi H.	2020	Y	Y	Y	Y	Y	U	Y						Y	N	Y	N	Y	^∗∗^
25	Sipilä S.	2021	Y	Y	Y	U	Y	Y	Y						Y	N	Y	N	Y	^∗∗^
26	Norouzi E.	2019	Y	Y	Y	Y	Y	N	Y						Y	U	Y	U	Y	^∗∗∗^
27	Rezola‐Pardo C.	2022	Y	Y	Y	Y	U	Y	Y						Y	Y	Y	U	Y	^∗∗∗∗^
28	Torre M. M.	2021	Y	Y	Y	U	U	U	U						Y	U	Y	N	Y	^∗∗^
29	Patti A.	2021	Y	Y	Y	Y	U	Y	U						Y	U	Y	U	Y	^∗∗∗^
30	Ferreira D. L.	2022	U	U	Y	U	U	U	Y						Y	U	Y	U	Y	^∗^
31	Carballeira E	2021	Y	Y	Y	U	U	U	Y						Y	U	Y	U	Y	^∗∗∗^

*Note:* Ratings were based on the number of “Yes” responses across relevant MMAT items.

^∗^MMAT scoring: = very low quality.

^∗∗^Low quality.

^∗∗∗^Moderate quality.

^∗∗∗∗^Good quality.

^∗∗∗∗∗^High quality.

This scoring mechanism facilitates a critical assessment of the overall quality of the evidence base.

### 2.6. Data Synthesis

Results were synthesized thematically and organized according to intervention type and outcomes reported (balance, gait, and cognitive function). Tables were used to present intervention characteristics and outcome trends.

## 3. Results

### 3.1. Search Results

**Figure 1:**
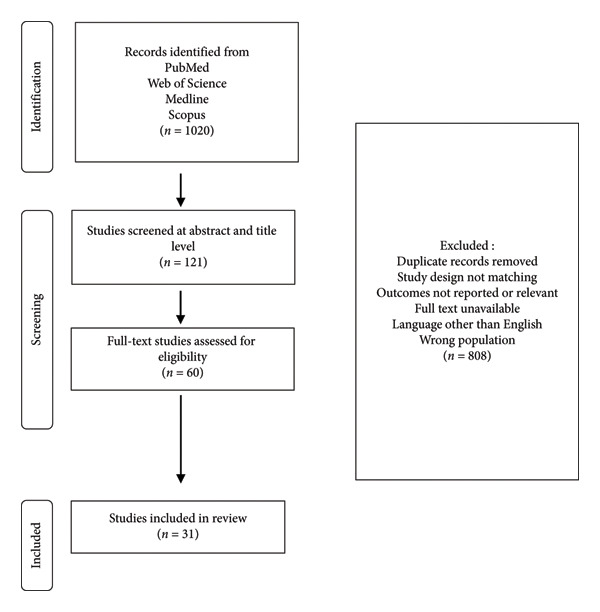
Flow diagram of article selection.

The comprehensive search strategy and study selection process employed in this scoping review are illustrated in Figure 1. Our scoping approach began with an extensive search across four major databases, PubMed, Web of Science, Medline, and Scopus, and search processes returned 1020 records, The selection process proceeded through multiple stages of rigorous screening. First, we conducted a preliminary assessment at the abstract and title level, which narrowed down to 121 potentially relevant studies. Subsequently, we performed a more in‐depth evaluation of 60 full‐text articles for eligibility against our predefined criteria. Throughout this process, we excluded 808 records based on various factors, including duplicate entries, misaligned study designs, irrelevant outcomes, inaccessible full texts, non‐English language publications, and studies focusing on populations outside our scope. The selection methodology culminated in the inclusion of 31 studies that form the main of our scoping review, ensuring a focused yet comprehensive examination of the current literature landscape on our topic of interest.

A total of 31 studies met the inclusion criteria, with participants being community‐dwelling older adults aged 60 and above. Intervention durations ranged from 6 weeks to 12 months with most studies implementing sessions 2‐3 times per week. Outcome measures commonly included assessments of balance, cognitive function, and gait performance.

Of the 32 included studies, 25 assessed balance outcomes, and 21 of these reported significant improvements.​ Gait‐related outcomes were evaluated in 22 studies, and 17 studies reported improvements in stride length, walking speed, or stability. Cognitive outcomes (e.g., executive function and memory) with 20 studies reported significant improvements.

Several studies reported that dual‐task interventions were associated with greater improvements in gait, dynamic balance, and cognitive function compared to single‐task interventions with motor–cognitive DTT appearing particularly effective in enhancing balance and mobility in older adults.

Participants being community‐dwelling older adults aged 60 and above. Intervention durations ranged from 6 weeks to 12 months with most studies implementing sessions 2‐3 times per week. Outcome measures commonly included assessments of balance, cognitive function, and gait performance. Several studies reported that dual‐task interventions were associated with greater improvements in gait, dynamic balance, and cognitive function compared to single‐task interventions with motor‐cognitive DTT appearing particularly effective in enhancing balance and mobility in older adults.

### 3.2. MMAT

Table 1 presents the MMAT. The analysis of 32 mixed‐methods studies in Table 1 reveals different levels of methodological quality. This comprehensive table evaluates research quality across multiple dimensions, including screening questions, RCTs, nonrandomized studies, and quantitative descriptive studies. The MMAT is designed to critically appraise the methodological quality of various study types within a single tool. Each study is assessed using a “Yes” (Y), “No” (N), or “Undefine” (U) rating system for various quality criteria specific to the study design. This scoring system provides a quick visual representation of each study’s methodological quality.

The Table 1 reveals considerable variability in study quality and completeness of data, with most studies passing initial screening questions but showing differences in their performance across specific methodological criteria. For instance, studies with ^∗∗∗∗∗^ ratings shows outstanding quality in across all assessed domains, while those with lower ratings may have areas for improvement in their study design. This MMAT scores provides valuable insights into the methodological strengths and limitations of the included studies, allowing a critical assessment of the overall quality of the evidence base in our scoping review. The MMAT’s comprehensive approach allows facilitating comparisons across different methodologies and highlighting areas where future research could strengthen its research design.

### 3.3. Characteristics of the Studies

Table 2 presents a comprehensive overview of 27 studies focusing on older adults, starting from 2014 to 2024 across different countries. 26 out of 27 of the studies are RCT design with one study using a quasi‐experimental approach. The sample populations primarily consisted of community‐dwelling older adults, with some studies specifically targeting sedentary or healthy older adults. The age ranges of participants varied, starting from 60 to 93 years. Most studies provided specific age ranges or means with standard deviations, while others used broader categories like above 60 or a range like 60–70 years.

**Table 2:** Characteristics of the studies.

No.	Author (year)	Country	Study design	Sample design	Age range
1	Wollesen et al. [12]	Germany	RCT	Community‐dwelling older adults	72.7 ± 4.7
2	Wollesen et al. [13]	Germany	RCT	Community‐dwelling older adults	71.5 ± 5.2
3	Yuzlu et al. [14]	Turkey	RCT	Community‐dwelling older adults	> 65
4	Piotrowska et al. [15]	Poland	RCT	Community‐dwelling older adults	64–93
5	Trombini‐Souza et al. [16]	Brazil	RCT	Community‐dwelling older adults	60–80
6	Nahand et al. [17]	Iran	QUASI‐EXPERIMENTAL	Sedentary older adults	63.57 ± 3.31
7	Jehu et al. [18]	Canada	RCT	Healthy older adults	60–77
8	Alves de Oliveira et al. [19]	Brazil	RCT	Community‐dwelling older adults	> 60
9	Javadpour et al. [20]	Iran	RCT	Community‐dwelling older adults	> 65
10	Park [21]	Korea	RCT	Healthy older adults	> 65
11	De Maio Nascimento et al. [22]	Brazil	RCT	Community‐dwelling older adults	66.20 ± 4.05
12	Ogawa et al. [23]	Boston	RCT	Community‐dwelling primary care patients	60–90
13	Jehu et al. [18]	Canada	RCT	Community‐dwelling older adults	67.0 ± 6.9
14	Castillo de Lima et al. [24]	Brazil	RCT	Community‐dwelling older adults	66.9 ± 5.0
15	Fraser et al. [25]	Canada	RCT	Sedentary older adults	> 60
16	Nayak et al. [26]	Canada	RCT	Community‐dwelling older adults	70–85
17	Sokołowska et al. [27]	Poland	RCT	Community‐dwelling older adults	73.8 ± 6.2
18	Eggenberger et al. [28]	Switzerland	RCT	Community‐dwelling older adults	> 70
19	Scarmagnan et al. [8]	Brazil	RCT	Sedentary older adults	68.5 ± 4.7
20	Akin et al. [10]	Istanbul	RCT	Community‐dwelling older adults	67.72 ± 7.33
21	Takeuchi et al. [29]	Japan	RCT	Community‐dwelling older adults	65:9 ± 13:7
22	Sipilä et al. [30]	Finland	RCT	Community‐dwelling older adults	70–85
23	Norouzi et al. [9]	Switzerland	RCT	Community‐dwelling older adults	> 65
24	Rezola‐Pardo et al. [31]	Spain	RCT	Community‐dwelling older adults	> 70
25	Torre et al. [32]	France	RCT	Independent‐living community dwellers	65–80
26	Smith‐Ray et al. [7]	United state	RCT	Community‐dwelling older adults	72.5
27	Ten Brinke et al. [33]	Canada	RCT	Community‐dwelling older adults	65–85
28	Blackwood et al. [34]	United state	RCT	Community‐dwelling older adults	> 70
29	Patti et al. [35]	Turkey	RCT	Healthy older adults	63.94 ± 4.37
30	Ferreira et al. [36]	Brazil	RCT	Community‐dwelling older adults	> 65
31	Carballeira et al. [37]	Spain	RCT	Healthy older adults	> 65

This Table 2 highlights the global nature of research on older adults, with studies conducted in diverse locations including European countries, North and South America, Asia, and the Middle East. There is consistency in study design and sample population comparable research in this field, while the variation in age ranges and specific sample characteristics indicates attention to different subgroups within the older adult population.

### 3.4. Interventions

For each included study, we extracted and categorized data regarding intervention characteristics, study design, and task type. Intervention data were organized into three categories: dual‐task interventions (Table A1), single‐task cognitive interventions (Table A2), and single‐task physical interventions (Table A3). For each study, we documented sample sizes, intervention protocols, duration and frequency of interventions, outcome measures, and follow‐up periods. This systematic approach allowed for comprehensive comparison across studies and identification of different intervention designs. Sample sizes range from 20 to 50 participants per group, with some larger studies including over 100 participants.

Interventions focused physical exercises to complex multicomponent programs. Physical interventions include balance training, Nordic walking, resistance training, and aerobic exercises. Cognitive interventions often involve tasks targeting executive function, memory, and attention. Most of the studies employ dual‐task or multicomponent interventions, combining physical and cognitive elements.

The duration and frequency of interventions vary considerably. Study lengths range from short‐term interventions of 6–8 weeks to long‐term programs lasting up to 12 months. Most of the studies are within the 8–24 weeks range. Session frequency is typically from 1 to 3 times per week, with sessions often lasting 45–60 min. This variability allows researchers to examine both immediate and sustained effects of different intervention intensities.

Outcome measures encompass a broad range of physical and cognitive assessments. Physical measures frequently include tests of balance (e.g., Berg Balance Scale), gait speed, strength (e.g., grip strength), and functional mobility (e.g., Timed Up and Go [TUG] test and 6‐min walk test). Cognitive assessments often feature tests of executive function, processing speed, counting number, and memory. Many studies also include measures of daily living activities and quality of life, providing a different view of intervention impacts.

Participant populations primarily consist of community‐dwelling older adults, typically starting from 60 and above. This targeted approach allows researchers to examine intervention effects on vulnerable populations within the broader older adult community.

Control conditions in these studies range from no‐intervention groups to those receiving different interventions or education programs. Control conditions help researchers differentiate between intervention effects.

Overall, these Tables A1, A2, A3 show a significant trend in geriatrics research towards integrating physical and cognitive training. This approach helps to understanding how physical and cognitive health impacts the aging populations and the potential of different interventions to improve benefits for older adults.

## 4. Discussion

The scoping review focuses on the effects of dual‐task and single‐task interventions on physical and cognitive functions in older adults, specifically targeting fall prevention, balance improvement, and gait performance. It highlights the challenges of aging, particularly in maintaining cognitive and motor functions, and explores intervention strategies to mitigate these issues. The review systematically examines studies that utilize DTT and single‐task interventions to enhance balance, cognitive functions, and reduce fall risks among older adults.

### 4.1. DTT

DTT is an intervention designed to address age‐related declines in motor and cognitive functions. It involves performing two tasks simultaneously, such as walking while remembering a list of words [8, 14]. Studies have shown that DTT provides benefits in areas like postural stability, mobility, and cognitive performance, with variables such as gait and speed frequently examined. For example, some studies specifically assess how DTT influences fall‐related factors like balance and walking stability, highlighting its potential in fall prevention. However, previous research often lacks consistency in variables and measures, which complicates comparisons across studies.

Most studies combine cognitive and physical tasks, such as walking while solving cognitive problems, while others focus on physical and physical combinations, such as resistance training with balance exercises. Some studies explore alternative approaches like sequential dual‐tasking, where participants switch between tasks, or simultaneous dual‐tasking, where tasks are performed together. For example, a pure DTT study focusing on simultaneous motor and cognitive tasks demonstrated significant improvements in walking speed and memory [9]. While pure DTT studies with larger sample sizes tend to have more reliable results, alternative dual‐task approaches show promise, but the sample sizes are small, which limits their validity.

A 12‐week DTT program focusing on both motor and cognitive tasks improved balance, walking functionality, and muscle strength in older adults, though it showed no significant effect on fear of falling [8, 10]. Interventions that integrate cognitive tasks into physical exercises appear particularly effective. For example, agility ladder training combined with cognitive tasks enhanced both physical and cognitive functions more than physical exercises alone [24].

When examining gait, DTT interventions have been shown to significantly improve walking smoothness and stability. Studies often use tasks like obstacle walking or treadmill‐based DTT, where participants engage in cognitive challenges while walking. Improvements in gait patterns are frequently observed after intervention periods of 12–24 weeks, with significant enhancements reported in dynamic balance and functional mobility [9].

DTT also improves walking speed, an important variable for fall prevention and daily functioning. In one study, a 10‐day DTT intervention led to small but noticeable increases in gait speed, whereas longer programs of up to 24 weeks demonstrated more substantial and lasting improvements [8]. These results indicate that both the duration and intensity of DTT influence its effectiveness in improving speed and reducing fall risk. Despite the promising evidence, research on DTT has notable gaps. A study comparing motor–cognitive DTT (mCdtt) and motor–motor DTT (mMdtt) acknowledged the lack of follow‐up as a limitation in assessing the durability [10]. The lack of standardization in secondary tasks further limits the comparability and generalizability of results across different studies. Future research should include follow‐up periods to assess the long‐term effects of DTT. This step is important for improving the interventions and ensuring they provide sustainable benefits for older adults.

In conclusion, DTT holds significant potential for addressing the unique challenges of aging. By improving both cognitive and physical functions, it can enhance mobility, stability, and overall well‐being in older adults. Addressing current research gaps will help develop evidence‐based programs that maximize the benefits of DTT for this population.

### 4.2. Cognitive Training

Cognitive training, particularly computerized cognitive training (CCT), has been identified as a promising approach to enhance cognitive and physical functions in older adults. CCT targets specific cognitive domains such as attention, processing speed, memory, and executive function. Executive function, which includes abilities like planning, task management, and integrating information, plays a key role in maintaining balance and preventing falls in older adults. Deficits in this area are strongly associated with an increased risk of falls, making it a critical target for intervention [34].

One study demonstrated that a CCT program focusing on attention and visuospatial ability significantly improved gait speed among participants, highlighting the link between cognitive improvements and physical outcomes [34]. Another study found that participants who underwent CCT performed better on the TUG test, a common measure of balance, compared to those in a control group. This effect was particularly notable among individuals categorized as slow walkers, suggesting that cognitive stimulation alone, without incorporating physical activities, can directly influence balance and mobility [7]. These findings underscore the importance of targeting cognitive processes, particularly memory and executive function, to support physical stability.

The effectiveness of CCT appears to depend on factors such as the duration and frequency of training, the specificity of the tasks, and individual differences among participants [33]. Combining CCT with aerobic exercise may further enhance its impact by improving the cognitive benefits of exercise‐induced brain plasticity. For example, engaging in physical activity immediately before a CCT session could see the improvements in attention and memory in older adults [33]. The benefits of CCT are also largely short‐term, as follow‐up studies to evaluate long‐term effectiveness are limited. Furthermore, variability in training protocols, such as differences in duration, task types, and intensity, makes it challenging to standardize and compare findings across studies. Additionally, while CCT can improve physical functions like gait and balance, these effects are indirect and may not match the benefits achieved through integrated cognitive–physical interventions.

In summary, single cognitive interventions like CCT can yield significant benefits in improving cognitive functions and associated physical outcomes, such as balance and gait speed. Despite its promise, addressing these limitations through more standardized protocols, long‐term studies, and diverse participant groups is essential for maximizing its effectiveness in fall prevention and functional improvement in older adults.

### 4.3. Physical Training

Single motor interventions have been shown to improve physical functions and, in some cases, cognitive abilities in older adults. These interventions often focus on specific exercises targeting balance, strength, and mobility. For instance, Pilates training and other general physical activities have been found to enhance postural control and hand grip strength, which are important for maintaining balance and reducing fall risks [35]. Similarly, low‐volume cycling programs have improved balance and gait performance, especially in older adults, suggesting that even moderate‐intensity exercises can benefit physical coordination and stability [37].

Most single‐motor interventions emphasize dynamic and functional exercises tailored to the participants’ needs. These include resistance training, walking exercises, and stretching routines. Land‐based interventions typically involve balance training and resistance exercises to address muscle weakness and improve stability during daily activities. Program durations vary, with most studies lasting between 6 and 24 weeks, with sessions conducted 2‐3 times per week, lasting 45–60 min each. Shorter programs, such as those lasting 10 days, show some immediate effects, but their long‐term impact is unclear.

One study conducted a 16‐week aquatic training program focusing solely on physical tasks. While not incorporating cognitive elements, this program effectively improved participants’ balance and motor performance, demonstrating the value of water‐based exercises in reducing the physical strain while enhancing motor functions [36]. Similarly, resistance training and aerobic exercises have shown consistent benefits in improving mobility, strength, and gait stability in older adults.

Despite their benefits, single‐motor interventions face limitations. The cognitive engagement in these interventions may limit their effectiveness in real‐life multitasking scenarios. Moreover, few studies include follow‐ups to evaluate the sustainability of these improvements over time. Standardizing protocols for single‐motor interventions could improve the comparability of research findings and their application in diverse older populations.

In conclusion, single‐motor interventions remain an effective approach for enhancing physical functions in older adults. They are particularly beneficial for addressing balance and mobility challenges, making them a valuable component of fall prevention strategies. Future studies should explore long‐term effects and refine intervention designs to maximize their practical benefits.

### 4.4. Cognitive, Physical, and Quality of Life Outcomes

The present study’s findings reveal different benefits of various training interventions in older adult populations. From a cognitive perspective, training programs have shown significant improvements in attention, processing speed, and working memory [7, 25]. Physical training, while focusing on exercise, has also unexpectedly enhanced cognitive functions, likely through mechanisms such as improved cerebral blood flow and stimulation of neural plasticity during aerobic exercises and complex motor activities [32]. Among these, DTT has emerged as particularly effective, enhancing executive function and attention by requiring individuals to manage multiple cognitive processes simultaneously [8, 22].

While the present study reveals comprehensive benefits, the formal evaluation of intervention efficacy based on varying subgroups, such as individuals with a history of falls or those presenting with baseline cognitive risk, remains limited due to the relatively general inclusion criteria employed in the primary studies. An initial observation across the included trials, particularly those targeting sedentary older adults, suggests that dual‐task and physical training are effective for this subgroup. Furthermore, studies such as Nahand et al. [17] demonstrated that DTT improved dynamic balance in older adults regardless of their cognitive status although quality of life gains appeared more stable in the high cognitive status group. Future research must prioritize trials focused on populations with specific conditions to advance the tailoring of interventions.

The physical benefits across these training programs are equally notable. Cognitive training, despite focusing on mental functions, unexpectedly improved balance and dual‐task gait speed [7]. Physical training consistently enhanced functional capacity and overall physical performance, including balance and gait, which are critical for independence [6, 37]. DTT further improved physical outcomes by enhanced postural stability, gait speed, and balance, highlighting its potential to optimize motor coordination through the integration of cognitive tasks [8, 18]. Though the direct impact on quality of life was less frequently assessed, the interventions showed promising indirect benefits, such as greater independence and social interaction. Cognitive training enhanced independence by strengthening mental capabilities like memory and executive functions [33], while physical training improved mobility and self‐confidence [37]. DTT, by simultaneously targeting cognitive and physical domains, significantly benefited older adults’ daily activities and social participation [18, 25]. Although the findings are positive, several limitations should be noted. Most studies involved healthy older adults limiting the understanding of intervention effects on populations with chronic conditions or mobility impairments. Short‐term effects were often measured, with limited follow‐up studies to assess the sustainability of benefits over time. Additionally, research exploring the neurophysiological mechanisms underlying these improvements is scarce. Tools like electroencephalogram (EEG) could help examine brain activity changes during training, while electromyography (EMG) could provide insights into muscle function and coordination.

Future studies should include follow‐up periods to assess long‐term effects and focus on populations with specific conditions to deepen understanding. Further exploration of the neurophysiological mechanisms behind these interventions could strengthen their design and applicability. Standardizing protocols and outcome measures would also help refine training approaches and maximize their benefits for older adults. By addressing these gaps, research can advance evidence‐based strategies to promote overall well‐being and functional independence in aging populations.

### 4.5. Implications for Future Research

Future research should include follow‐up periods to assess long‐term effects and focus on populations with certain conditions to deepen understanding [8]. Further exploration of the neurophysiological mechanisms behind these interventions could strengthen their design and applicability. Tools like EEG could help examine brain activity changes during training, while EMG could provide insights into muscle function and coordination [22]. Standardizing protocols and outcome measures would also help refine training approaches and maximize their benefits for older adults [18]. Correspondingly, future research should prioritize the reporting of effect sizes and minimum clinically important differences (MCID) alongside statistical significance to ensure that findings are clinically relevant and readily applicable in practice. By addressing these gaps, research can advance evidence‐based strategies to promote overall well‐being and independence in aging populations.

### 4.6. Implications for Clinical Practice

The scoping review highlights the effectiveness of dual‐task, cognitive, and physical training interventions in improving balance, mobility, and cognitive function in older adults [24]. These findings suggest that healthcare professionals should incorporate DTT into rehabilitation programs, particularly for fall prevention and cognitive–motor integration [10]. Physical therapists can integrate these interventions into structured programs to enhance functional independence in older adults [8]. Additionally, cognitive–motor training should be tailored to individual needs, ensuring that interventions can be tailored to different levels of physical and cognitive ability.

### 4.7. Strengths and Limitations of the Study

Strength of this review is its comprehensive examination of various interventions, allowing a clear comparison of their effectiveness in improving cognitive and physical outcomes [9]. Additionally, the inclusion of a wide range of study designs provides a broad perspective on intervention strategies [25].

### 4.8. Limitations

This scoping review has several limitations. First, the inclusion of only RCTs published in English may have excluded relevant studies published in other languages or using other designs potentially limiting the comprehensiveness of the review. Second, although the MMAT was used to assess methodological quality, this was not used to exclude any studies, and thus some included trials may have notable biases. Third, the review did not assess long‐term intervention outcomes or follow‐up data, as many included studies lacked this information. Fourth, the review did not assess the effect size or the MCID of the interventions. This primarily due to the inconsistent reporting of these metrics within the primary included studies. This represents a significant methodological limitation as the clinical relevance and practical utility of the observed statistical improvements remain challenging to interpret without these standardized measures. Lastly, publication bias may be present, as unpublished studies and gray literature were not included.

### 4.9. Funding

This study received no specific grant from any funding agency in the public, commercial, or not‐for‐profit sectors. The authors conducted this review as part of academic requirements and institutional research development.

## 5. Conclusion

This scoping review highlights the effectiveness of dual‐task, cognitive, and physical training interventions in enhancing functional capabilities and quality of life among the older adults. DTT emerges as particularly promising, addressing both cognitive and physical domains to improve postural stability, mobility, and memory. Cognitive training enhances executive function and balance, while physical training contributes to cognitive performance and dual‐task capabilities.

Future research must prioritize trials focused on subgroups (e.g., those with fall risk) and ensure the reporting of effect sizes and MCID to maximize the clinical relevance and sustainability of benefits for aging populations.

## Conflicts of Interest

The authors declare no conflicts of interest.

## Funding

No funding was received for this manuscript.

## Data Availability

The data that support the findings of this study are available from the corresponding author upon reasonable request.

## Appendix A

**Table A1:** Dual‐task interventions.

No.	Study	Group	Intervention	Duration and frequency	Follow‐up	Measure outcomes	Task type	Outcome/key findings
1	Wollesen et al. [12]	Intervention (*n* = 19)	Phase 1: Training daily actions with high fall risk. Phase 2: Task prioritization, task switching, and transfer.	12 weeks, 1 session/week, 60–90 min/session	No	Gait performance, maximum forces, dual‐task costs	DT	Gait performance ↑ Maximum forces ↓ Dual‐task costs ↓
		Control (*n* = 19)	No intervention	12 weeks, 1 session/week, 60–90 min/session	No	Gait performance, maximum forces	ST	Gait performance ↑ Maximum forces ↓

2	Wollesen et al. [13]	DT training no COF^∗^ (*n* = 26)	DT training (Phase 1: daily actions with high fall risk; Phase 2: task prioritization, task switching).	12 weeks, 1 session/week, 60 min/session	No	Step length, step width, gait line, cognitive, foot Rolling	DT	Step length ↑ Step width ⟶ Gait line ↑ Cognitive ↓ Foot rolling ↑
		DT training COF (*n* = 26)	DT training (Phase 1: daily actions with high fall risk; Phase 2: task prioritization, task switching).	12 weeks, 1 session/week, 60 min/session	No	Step length, step width, gait line, cognitive, foot rolling	DT	Step length ⟶ Step width ⟶ Gait line ⟶ Cognitive ⟶ Foot rolling ⟶
		CG (*n* = 19)	Control group: no intervention	12 weeks, 1 session/week, 60 min/session	No	Step length, step width, gait line, cognitive, foot rolling	ST	Step length ⟶ Step width ⟶ Gait line ⟶ Cognitive ⟶ Foot rolling ⟶
		CG COF (*n* = 19)	Control group: no intervention	12 weeks, 1 session/week, 60 min/session	No	Step length, step width, gait line, cognitive, foot rolling	ST	Step length ↓ Step width ⟶ Gait lline ↓ Cognitive ↓ Foot rolling ↓

3	Yuzlu et al. [14]	Dual‐task training (IDTT) (*n* = 29)	Balance exercises performed simultaneously with cognitive tasks	8 weeks, 2 sessions/week	No	BBS, TUG‐ST, TUG‐Cog, FES, 10MWT‐ST, 10MWT‐DT	DT	BBS ↑ TUG‐ST ↑ TUG‐Cog ↑ FES ↑ 10MWT‐ST ⟶ 10MWT‐DT ↑
		Consecutive dual‐task (CDTT) (*n* = 29)	Balance exercises and cognitive tasks performed separately (consecutively)	8 weeks, 2 sessions/week	No	BBS, TUG‐ST, TUG‐Cog, FES, 10MWT‐ST, 10MWT‐DT	DT	BBS ↑ TUG‐ST ↑ TUG‐Cog ↑ FES ↑ 10MWT‐ST ⟶ 10MWT‐DT ↑

4	Piotrowska et al. [15]	Nw combined with ct (*n* = 20)	Nordic walking, strength/flexibility exercise with arithmetic problems	3 months, 2 sessions/week, 60 min/session	No	Left/right leg static balance, agility, hand strength	DT	Left leg ↑ Right leg ↑ Agility ↑ Hand strength ↑
		Nw (*n* = 20)	Nordic walking, strength exercise, breathing and flexibility exercise.	3 months, 2 sessions/week, 60 min/session	No	Left/right leg static balance, agility, hand strength	ST	Left leg ↑ Right leg ↑ Agility ↑ Hand strength ↑
		Cg (*n* = 21)	No intervention	3 months, 2 sessions/week, 60 min/session	No	Left/right leg static balance, agility, hand strength	ST	Left leg ⟶ Right leg ⟶ Agility ⟶ Hand strength ⟶

5	Trombini‐Souza et al. [16]	Experimental group (EG) (*N* = 30)	Stage 1 (1–12 weeks): alternates single motor task and simultaneous dual task. Stage 2 (13–24 weeks): strictly simultaneous dual task	24 weeks, 4 stations/session, 10 min each	12 weeks	SDT, gait swing phase, double support phase, TUG conventional/cognitive, dual‐task cost TUG, static/dynamic balance, body sway, TMT‐A/B, stroop test/cost	DT	SDT ↑ Gait swing phase ↑ Double support phase ↓ TUG conv/cog ↑ Dual‐task cost TUG ↑ Bal/Post ↑ ↓ Bodysway ↑ (open/closed) TMT‐A/B ↑ Stroop test/cost ↑

6	Nahand et al. [17]	Low cognitive status (*n* = 10)	Aquatics exercise with cognitive training (braingym)	6 weeks, 3 sessions/week	No	QoL, fall self‐efficacy, static/dynamic balance (Romberg/TUG)	DT	QoL ↑ Fall self‐efficacy ↑ Static balance ↑ Dynamic balance ↑
		High cognitive status (*n* = 10)	Aquatics exercise with cognitive training (braingym)	6 weeks, 3 sessions/week	No	QoL, fall self‐efficacy, static/dynamic balance (Romberg/TUG)	DT	QoL ⟶ Fall self‐efficacy ↑ Static balance ↑ Dynamic balance ↑

7	Jehu et al. [18] (12 weeks)	BMT + C group (*n* = 17)	Static and dynamic exercise with obstacle + cognitive task	12 weeks, 3 sessions/week, 1 h/session	12 weeks	COP area/velocity/entropy, reaction time	DT	Postural Sway ⟶ Reaction time ↑
		BMT (*n* = 17)	Static and dynamic exercises with obstacle	12 weeks, 3 sessions/week, 1 h/session	12 weeks	COP area/velocity/entropy, reaction time	ST	Postural Sway ⟶ Reaction time ↑

8	Alves de Oliveira et al. [19]	C + UST (*n* = 25)	Cognitive training simultaneously with unstable strength training	24 weeks, 3 sessions/week, 60 min/session	No	TUG, TUGcog, DTC, BBS, Sit‐and‐reach, FES, QoL	DT	TUG ↑ TUGcog ↑ DTC ↑ BBS ↑ Sit‐and‐reach ↑ FES ↑ QoL ↑
		UST (*n* = 25)	Unstable strength‐training	24 weeks, 3 sessions/week, 60 min/session	No	TUG, TUGcog, DTC, BBS, Sit‐and‐reach, FES, QoL	ST	TUG ↑ TUGcog ⟶ DTC ⟶ BBS ↑ Sit‐and‐reach ↑ FES ↑ QoL ↑

9	Javadpour et al. [20]	Dual task group (*n* = 23)	Balance exercises with cognitive task	6 weeks, 18 sessions	No	Gait smoothness (HR), gait speed, FAB scale, ABC scale	DT	Gait smoothness ↑ Gait speed ↑ FAB scale ↑ ABC scale ↑

10	Park [21]	Experimental group (EG) (*N* = 29)	Balance tasks with cognitive tasks	6 weeks, 12 sessions	No	Static balance (OLST), dynamic balance (TUG), executive function (TMT‐B)	DT	Static Balance ↑ Dynamic balance ↑ Executive function ↑

11	De Maio Nascimento et al. [22]	Dual task group (*N* = 25)	Physical–cognitive dual‐task training	24 weeks, 2 sessions/week	12 weeks	MMSE, verbal fluency tests, TUG conventional/manual/cognitive, TEC‐body balance test, lower extremity strength	DT	MMSE ↑ Verbal fluency ↑ TUG conv/m/c ↑ Bal/strength ↑ Maintenance ↑

12	Ogawa et al. [23]	Invest + TAPAT (*N* = 11)	Computerized cognitive training and progressive resistance training exercise	6 weeks, 3 sessions/week	No	Leg power, dynamic balance (Figure‐of‐8), SPPB, gait speed, step length, stance time/variability, swing time variability	DT	Leg power ↑ Dynamic balance ↑ SPPB ↑ Gait speed ↑ Step length ↑ Stance/swing time Variability ⟶

13	Jehu et al. [18] (24 weeks)	BMT + C group (*n* = 17)	Static and dynamic exercise with obstacle + cognitive task	24 weeks, 3 sessions/week, 1 h/session	12 weeks	TUG, TUG cognitive, TUG manual, test stability	DT	TUG ↑ TUG cognitive ↑ TUG manual ↑ Test stability $\rightarrow$26

14	Castillo de Lima et al. [24]	AT + CT group (*N* = 14)	Agility training with cognitive task	16 weeks (2 weeks familiarization + 14 weeks training)	No	TUG‐cog, phonological VF, SPMT, walking speed (WS), muscle power (5XSTS), dynamic balance (TUG), handgrip strength (IHG), agility function (IAT)	DT	TUG‐cog ↑ Phonological VF ↑ SPMT ↑ WS ↑ Power ↑ TUG ↑ IHG ↑ IAT ↑

15	Fraser et al. [25]	Aerobic + CT (*N* = 21)	Aerobic exercise with cognitive training	12 weeks, 1 cognitive/week, 2 physical/week	No	Dual‐task walking (DT‐walk), dual‐task balance (DT‐balance), cognitive accuracy	DT	DT‐walk ↑ DT‐balance ↑ Cognitive accuracy ↑
		Stretch + CT (*N* = 18)	Stretching exercise with cognitive training	12 weeks, 1 cognitive/week, 2 physical/week	No	Dual‐task walking (DT‐walk), dual‐task balance (DT‐balance), cognitive accuracy	DT	DT‐walk ↑ DT‐balance ↑ Cognitive accuracy ↑

16	Nayak et al. [26]	DT‐TR (*n* = 11)	Balance and walking exercise with cognitive task	10 weeks, 2 sessions/week, 45 min/session	No	Overall gait, TMT‐A/B, verbal fluency, standing balance, visuomotor/visuospatial executive function, game performance	DT	Overall gait ↑ TMT‐A/B ↑ Verbal fluency ⟶ Standing balance ↑ Visuomotor/visuospatial ↑ Game perf ↑
		DT‐RC (*n* = 11)	Cycling exercise with cognitive task	10 weeks, 2 sessions/week, 45 min/session	No	Overall gait, TMT‐A/B, verbal fluency, standing balance, visuomotor/visuospatial executive function, game performance	DT	Overall gait ⟶ TMT‐A/B ↑ Verbal fluency ⟶ Standing balance ↑ Visuomotor/visuospatial ↑ Game perf ↑

17	Sokołowska et al. [27]	VR BCT (*N* = 18)	Balance with cognitive training	10 days, 30 min/session	No	TUG, FTSS, Romberg test (EO/EC), COP path length/velocity/area	DT	TUG ↑ FTSS ↑ Romberg test (EO/EC) ↑ COP path/vel/area ↑

18	Eggenberger et al. [28]	DANCE (*N* = 30)	Cognitive with physical training	6 months, 2 sessions/week, 1 h/session	3, 6, 12 months	TMT‐B, executive control task, other cognitive tasks, PACES score	DT	TMT‐B ↑ Executive control ↑ Other cognitive ⟶ PACES ↑
		MEMORY (*N* = 29)	Treadmill walking with verbal memory exercise	6 months, 2 sessions/week, 1 h/session	3, 6, 12 months	TMT‐B, executive control task, other cognitive tasks, PACES score	DT	TMT‐B ↑ Executive control (initial ↑, then ↓) Other cognitive⟶ PACES ⟶

19	Scarmagnan et al. [8]	Experimental group (EG) (*N* = 24)	Strength, stretching, walking exercise with cognitive task	3 months, 2 sessions/week, 60 min/session	No	Postural stability, mobility (get‐up‐and‐go test), cognitive function, falls efficacy scale	DT	Postural stability ↑ Mobility ↑ Cognitive function ↑ Falls efficacy scale ⟶

20	Akin et al. [10]	Motor–cognitive DTT (mCdtt) (*N* = 25)	Functional task with cognitive task	8 weeks, 40 min/session	No	Balance performance (BBS), Fear of falling (FES‐I), walking functionality (TUG), muscle strength	DT	BBS ↑ (within), TUG ↑ (within) FES‐I ⟶ (within) Muscle strength ↑ (indirect)
		Motor–motor DTT (mMdtt) (*N* = 25)	Functional task upper limb and lower limb (two motor tasks)	8 weeks, 40 min/session	No	Balance performance (BBS), Fear of falling (FES‐I), walking functionality (TUG), muscle strength	DT	BBS ↑ (within) TUG ⟶ (within) FES‐I ⟶ (within) Muscle strength ↑ (indirect)

21	Takeuchi et al. [29]	SDAEWMT (*N* = 32)	Simultaneous dual‐task and aerobic exercise with WMT	12 weeks, 3 sessions/week	No	Cognitive functions, working memory (fMRI)	DT	Cognitive functions ⟶ Working memory (fMRI) ↑

22	Norouzi et al. [9]	Motor–cognitive DTT (mCdtt) (*N* = 20)	Resistance training with cognitive tasks	12 weeks, 1 session/week	No	Working memory, balance (BBS), time × group interaction	DT	Working memory ↑ Balance (BBS) ↑ Time × group interaction ↑
		Motor–motor DTT (mMdtt) (*N* = 20)	Resistance training with simultaneous motor skills	12 weeks, 1 session/week	No	Working memory, balance (BBS), time × group interaction	DT	Working memory ↑ Balance (BBS) ↑ Time × group interaction ↑

23	Sipilä et al. [30]	Dual training (PT)	Targets physical and cognitive training sessions twice a week	6 months, 4 sessions/week	No	TMT A, dual‐task gait speed, stroop color and word test, gait speed	DT (P + C)	Addition of cognitive training (PCT) did not provide additional benefits over physical training alone. Overall, outcomes compared to physical training alone ↑
		Control (PCT)	Included the physical training program and the cognitive training session	6 months, 4 sessions/week	No	TMT A, dual‐task gait speed, stroop color and word test, gait speed	DT (P + C)	Both groups increased TUG velocity under cognitive tasks, but ⟶ significant differences between them

24	Rezola‐Pardo et al. [31]	Multicomponent dual‐task group	Simultaneous cognitive training applied to 8 out of 16 resistance and balance exercises	5 months, 2 sessions/week	12 months	TUG velocity, fall rate, Kaplan–Meier analysis	DT	Lower fall rate ↓ and significantly greater TUG/walking capacity ↑ compared to multicomponent group during intervention and 12‐month follow‐up

25	Torre et al. [32]	CT‐c/CT‐fit	Conventional circuit training/fitness gaming circuit training (both with cognitive tasks)	Protocol: 12 weeks, 2 sessions/week, 60 min/session	No	MoCA, TMT, Stroop task, dual‐task, gait assessments, TUG, unipedal/tandem balance, strength	DT	(Study Protocol) Hypothesized CT‐fit would be more effective in improving both cognitive and physical functions ↑ than traditional methods
		NW (nordic walking)	Nordic walking (physical/motor focus)	Protocol: 12 weeks, 2 sessions/week, 60 min/session	No	MoCA, TMT, Stroop task, dual‐task, gait assessments, TUG, unipedal/tandem balance, strength	ST (motor)	(Study Protocol) Expected NW to be outperformed by CT groups in walking speed and balance

**Table A2:** Single‐task interventions (cognitive).

No.	Study	Group	Intervention	Duration and frequency	Follow‐ up	Measure outcomes	Task type	Outcome/key findings
1	Smith‐Ray et al. [7]	Intervention group (*n* = 22)	Computer‐based training focusing on executive functions.	10 weeks, 2 times per week (60 min/session)	No	Berg Balance Scale (BBS), 10‐m gait speed (normal/dual‐task visuospatial)	ST (cognitive)	Balance (BBS) ↑ Normal Gait Speed ↑ Dual‐Task Gait Speed ↑

2	Ten Brinke et al. [33]	Fit brains training (FBT) (*n* = 41)	Computerized cognitive training.	8 weeks, 3 sessions/week, 60 min/session	No	Verbal memory (RAVLT), executive function	ST (cognitive)	Verbal Memory ⟶ Executive Function ↑

3	Blackwood et al. [34]	Intervention group (*n* = 24)	Computer‐based cognitive training.	6 weeks, 3 times per week, 20–25 min/session	No	Cognitive performance, gait speed, five times sit to stand (FTSTS), timed up and go (TUG)	ST (cognitive)	Cognitive Performance ⟶ Gait Speed ↑ FTSTS ⟶ TUG ⟶

**Table A3:** Single‐task interventions (physical).

No.	Study	Group	Intervention	Duration and frequency	Follow‐ up	Measure outcomes	Task type	Outcome/key findings
1	Patti et al. [35]	Pilates group (P‐G) (*n* = 23)	Pilates training.	13 weeks, 3 sessions/week	No	Berg Balance Scale, hand grip test, ellipse surface, postural control	ST (physical)	Hand Grip ↑ Ellipse Surface ↓ Postural Control ↑

2	Ferreira et al. [36]	Aquatic training group (ATG) (*n* = 24)	Multicomponent aquatic training.	16 weeks, 2 sessions/week, 1 h/session	No	5‐times sit‐to‐stand test, TUG, TUG‐DT, postural stability	ST (physical)	Muscle Strength & Mobility ↑ Postural Stability ⟶ Cognitive‐Motor Performance ↑

3	Carballeira et al. [37]	Exercise group (EG) (*n* = 12)	Motorized cycle ergometer	6 weeks, 60 min/week (18 sessions)	No	Body composition, short physical performance battery (SPPB), POMA‐T, hemodynamic parameters	ST (physical)	Waist Circumference ↓ Hemodynamic Parameters (SBPrest) ↓ Functional Performance ⟶
